# A blockchain-based smart healthcare system for data protection

**DOI:** 10.1016/j.isci.2025.112109

**Published:** 2025-03-03

**Authors:** Jide Kehinde Adeniyi, Sunday Adeola Ajagbe, Abidemi Emmanuel Adeniyi, Korede Israel Adeyanju, Adenrele A. Afolorunso, Matthew O. Adigun, Isaac Ogene

**Affiliations:** 1 Department of Computer Science, Landmark University, Omu-Aran, University SDG 4 (Quality Education), Omu-Aran, Nigeria; 2 Department of Computer Science, University of Zululand, Kwadlangezwa 3886, South Africa; 3 Department of Computer Engineering, Abiola Ajimobi Technical University, Ibadan 200255, Nigeria; 4 College of Computing & Communication Studies, Bowen University, Iwo, Nigeria; 5 Chitkara University Institute of Engineering and Technology Chitkara University, Punjab, India; 6 Department of Computing, Sheffield Hallam University, Sheffield S1 1WB, UK; 7 Department of Computer Science, National Open University of Nigeria, Abuja 900001, Nigeria

**Keywords:** Bioinformatics, Computer science

## Abstract

The security of medical information has become a significant challenge with the move from traditional filing systems to electronic records. This study proposes the use of blockchain technology to address these concerns. The system registers patients and medical staff with unique IDs and stores patient diagnoses as immutable records on the blockchain. A central interplanetary file system stores the collected data, which can be accessed by authorized users like nurses, pharmacists, and patients via special access details. Users must log in before accessing medical records through the Electronic Record Management system. This approach can be scaled to multiple hospitals. After testing, the system showed some latency issues with 100 nodes, but performance improved with more nodes (300–500), demonstrating better scalability as the system handles more data and hospitals. Overall, the proposed blockchain-based system offers a secure, scalable solution for managing and accessing medical records.

## Highlights

• Centralized IPFS for hospital HRMs
• Efficient information management
• Efficient Integration

## Graphical abstract

**Figure:**
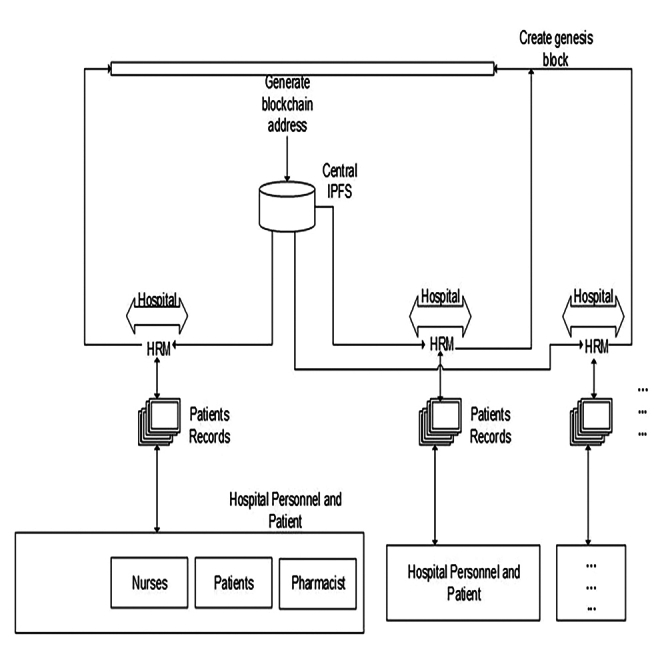

## Introduction

Even with the subsequent advancement in network security, hackers and data sniffers are still lurking around our network looking for important data they can steal and sell on the dark web.^1^^,^^2^^,^^3^^,^^4^ Through observation and survey, it has been noticed that a lot of millions has been lost in customers data theft. With losses estimated at over $560 million, the Federal Bureau of Investigation (FBI) reports receiving over 330,000 reports of identity theft. A $1 trillion estimate of company losses was also released by McAfee. Regrettably, this percentage will probably increase as Trojans, which are capable of stealing user information, make up 72% of newly discovered malware.^5^^,^^6^^,^^7^ An unlawful entry into your company’s network or a specific machine address in your designated domain is a network intrusion. There are two types of intrusions: passive (when the infiltration is acquired covertly and undetected) and active (in which changes to network resources are affected).

Both internal and external intrusions into your network are possible (by an employee, a customer, or business partner). Other incursions are merely intended to alert you to their presence by defacing your website with offensive text or graphics. Others, who have a more sinister intent, are out to steal important data, either once and for all or as part of a continuous parasitic relationship that siphons off information until it is detected.^8^^,^^9^ The traditional acronym CIA serves as a concise summary of the three fundamental principles of information security. This represents availability, integrity, and confidentiality. Studying information security entails studying the "CIA."^7^ Confidentiality is the act of keeping something private confidential. Cryptography, access control, and other methods are used to keep information hidden. Information security integrity means that data are not changed without the necessary procedure and authorization, whether on purpose or accidently. Availability denotes that the systems are functioning and useable as intended.^10^ A notable system where data are stolen is the healthcare system.

Data in a healthcare system that are usually stolen include patient’s health records, insurance information, addresses, social security number and card payment information, and so on. To prevent intrusion, intrusion prevention system is used.^11^^,^^12^ A type of network security called an intrusion prevention system (IPS) tries to identify threats and stop them from happening. Systems for preventing intrusions continuously scan your network for any hostile activities and record information about them.^9^^,^^13^ The IPS notifies system administrators of these occurrences and takes corrective action, such as shutting down access points and setting up firewalls to block further intrusions.^14^^,^^15^^,^^16^ A current technology used in intrusion prevention system is the blockchain technology.^17^^,^^18^^,^^19^

Even though intrusion detection systems have been developed to curb intrusions, not all intrusions can be detected before it becomes catastrophic. To reduce the rate at which data are stolen and networks are being hacked, there is a need to design and develop an intrusion prevention system using a blockchain network system that will help manage the intrusion on a system’s network.^20^^,^^21^ The intrusion prevention system would prevent hackers from gaining access to information on a network, because it would be almost impossible to gain control of over 50% of the blockchain system to make changes to the blocks and take out data.^22^ In this study, a data protection system as a means of intrusion prevention is presented for healthcare institutions using blockchain technology. Implementing a data protection system for a smart healthcare-based system using blockchain technology offers several advantages: enhanced security and privacy, time management, patient-centric data control, interoperability and standardization, data integrity and reliability, efficient data management, and improved patient control and consent management.

This study tends to provide numerous benefits for patients, healthcare providers, and organizations alike. The contribution of this study includes the following:

Protecting sensitive data: healthcare systems deal with sensitive and personal information of patients. The data must be secure and protected from unauthorized access or misuse. With blockchain technology, data can be encrypted and securely stored, making it virtually impossible for unauthorized access.

Ensuring data integrity: the integrity of data is critical in the healthcare industry. Any manipulation or alteration of data can have serious consequences. Blockchain technology provides a tamper-proof data storage mechanism, ensuring that data remain accurate and unaltered.

Enhancing interoperability: smart healthcare systems are built on a variety of technologies and platforms, which can make it challenging to exchange data between them. Blockchain technology can provide a secure and decentralized platform for data sharing, allowing various systems to interoperate seamlessly.

Improving patient outcomes: a smart healthcare system based on blockchain technology can improve patient outcomes by ensuring that the right data are available to the right people at the right time. This can help clinicians make better-informed decisions and improve the quality of care.

Compliance with regulations: the healthcare industry is heavily regulated, and organizations must comply with various regulations and standards to ensure patient privacy and data protection. Implementing a data protection system using blockchain technology can help organizations meet regulatory requirements.

This study consists of five sections. The next section describes the literature reviews. The methodology was described in section STAR Methods. Section results and discussion presents the result and discussion, whereas section conclusion concludes the study.

### Literature review

Block chain has been applied to several systems for data protection and integrity.^23^^,^^24^^,^^25^ Among the literatures are the study of ^24^ Akash and Ferdous^24^ that proposed a blockchain based system for healthcare digital twin. The technology called a "digital twin" (DT) may transfer any physical occurrence from a physical space to a digital realm while maintaining physical consistency. The system examined an approach to solve the privacy and security challenge in healthcare. According to the study, the correct method of obtaining structured data and securely keeping it is crucial due to the present research gaps. Their study presented a mathematical data model that allows for the organized and specified collection of pertinent patient data with appropriate demarcation. Furthermore, the description of the given data model aligns with real-world scenarios.

According to Ismail et al.,^26^ the client-server architecture used to store Electronic Health Records (EHRs) currently permits hospitals or cloud service providers to maintain stewardship of patient data. Furthermore, heterogeneous databases are used to disperse patient records around several hospitals. As a result, patients struggle to put together a coherent picture of their medical history so they can concentrate on the specifics of their treatment. The blockchain’s security characteristics and replication mechanism have a bright future in the medical field; hence, this study presented a blockchain-based framework for health records management (BlockHR), which gives medical practitioners access to a medical support system for improved patient follow-up and diagnosis. BlockHR has features that allowed users to upload lifestyle and medical information in order to estimate their chance of contracting chronic illnesses. The study selected Hyperledger Fabric, a permissioned channel-based blockchain technology that enables private transactions between subsets of network users, to build the architecture for health-care assistance. Because of the permission-less network’s shortcomings, including sluggish network performance and unwanted involvement, the permissioned network was preferred. In order to protect patient privacy, the channel-based design limited access to the medical data to a subset of approved hospitals within the network. In Hyperledger Fabric, the blockchain network was made up of users, assets, transactions, and events.

Chen et al.^27^ proposed a blockchain-based electronic medical record (EMR) sharing system for inter-hospital application. In the study, a smart contract was used. Mutual authentication was used to achieve data integrity, non-repudiation, user untraceability, and other features. To achieve inter-hospital access to patient medical records, a central blockchain was proposed to be maintained by the government. In their system, the communication between the hospitals and the blockchain was secured but that of the patient with the hospital was not. The efficiency of communication between each node in the system showed 0.181 ms for patient or hospital registration on a 3.5G network, 0.025 ms on a 4G network, and ol126 us on a 5G network. The system recorded 0.201 ms, 0.028 ms, and 0.141 us for 3.5G, 4G, and 5G, respectively.

In the study of Hang et al.,^28^ they proposed a smart contract-based blockchain-based medical platform to secure EMR administration. With this approach, patients may easily access their medical records from various hospital departments and receive a comprehensive, unchangeable log. A permissioned network was used to build a case study for a hospital, and a number of experimental tests are run to show the effectiveness and usefulness of the platform. In their system, medical equipment that were connected to the Internet of Things can send data continuously. These data were valuable for data analytics, which in turn generated a range of services, including critical care response and preventive care. IoT data exchange instantaneously allowed healthcare practitioners to provide faster and more accurate patient treatment. Their system produced a query transaction time of 56.6 ms, 56.1 ms, 58.7 ms, and 56.9 ms for 50 users, 250 users, 500 users, and 1,000 users, respectively. The invoke transaction had an average of 2710 ms, 2709 ms, 2820 ms, and 2984 ms for 50 users, 250 users, 500 users, and 1,000 users, respectively. The average latency ranged from 7.22 to 13.97 s for an invoke operation and has a throughput range of 430 to 500 tps. A query transaction had an average latency of 0.20s–23.19 s and a throughput range of 1,000 tps to 1,090 tps.

Due to the rise of internet pharmacies, it has proven difficult to detect counterfeits. Hence, Jamil et al.^29^ proposed a supply chain for drugs using the blockchain technology for smart hospitals. In this work, they presented a new approach to medication supply chain management that handles safe drug supply chain data using Hyperledger Fabric, a blockchain-based platform. By executing drug record transactions on a blockchain to build a smart healthcare ecosystem with a drug supply chain, the suggested method addresses this issue. To allow for time-limited access to patient electronic health information and electronic medication records, a smart contract was introduced. The result obtained showed an average latency of 154 ms for 100 users, 172 ms for 300 users, and a latency of 436 ms for 500 users.

Ismail et al.^30^ suggested a lightweight blockchain for the healthcare data management that has less computational and communication overhead than that of the Bitcoin network by dividing the network participants into clusters that maintained only a copy of the ledger in each cluster. The conclusion drawn from their architecture is the inclusion of the canal, and this ensures that special and sensitive transactions can be done within a network of participants. In addition, they also wanted to prevent forking, something that is particularly characteristic of the Bitcoin network. Their system showed a processing time of around 2.3 s, 2.7 s, and 2.85 s for 100 nodes, 300 nodes, and 500 nodes, respectively.

Cao et al.^31^ suggested a better algorithm based on Two_Arch2 to increase the blockchain’s scalability and decentralization while lowering its latency and cost. A multi-objective blockchain-enabled IIoT model was created by incorporating the private blockchain theory into IIoT while also considering private blockchains with decentralization, adaptable regulations, and strong privacy protection goals. Then, the model was solved using an enhanced Two_Arch2 algorithm. The enhanced method can efficiently optimize the four model indicators, according to experimental findings. MOEA/D performs better than the other three algorithms in optimizing the scalability; however, it obtains the worst results in time to finality (TTF), decentralization, cost and execution time was not considered unlike the current study.

Xie et al.^32^ attempted to make privacy protection on devices more palatable locally by lowering the requirements for hostile sample privacy safeguards. Adversarial-sample-based privacy measures rely on deep learning (DL) models, which can be difficult to deploy due to their enormous number of parameters. Thankfully, a method called model structural pruning has been put forth that can be used to lower the number of parameters in DL models. The study created two structural-pruning-based adversarial sample privacy protections, where the user accesses the perturbed data through the pruned DL model. These are based on the model pruning approach DepGraph and the currently available adversarial sample privacy protections AttriGuard and MemGuard. The do extensive experiments on four datasets, and the findings show adversarial sample privacy protection based on structural pruning was effective. However, the study falls short of data management.

He et al.^33^ proposed a novel Dynamic-Graph-Transformer-based Parallel Framework (DGT-PF) in order to more effectively detect system anomalies in cloud infrastructures. This framework used graph neural network (GNN) to learn the spatiotemporal features of KPIs and Transformer with anomaly attention mechanism to improve the accuracy and timeliness of model anomaly detection. More specifically, it was suggested to use an efficient dynamic relationship embedding technique to soft cluster each GNN layer using the Diffpooling module, dynamically learn spatiotemporal characteristics, and adaptively create adjacency matrices. Furthermore, the authors employed both the AR-MLP model and the nonlinear neural network model in tandem to enhance detection performance and achieve higher detection accuracy. According to the experiment, out of 11 anomaly detection models, the DGT-PF framework had the greatest F1-Score on five public datasets, with an average improvement of 21.6%. The study failed to consider the latency and execution time, which are key components in data security and management scenario.

## Results and discussion

The implementation details of the data protection system using blockchain is presented here. It contains the software and hardware requirements for the blockchain system. It shows the steps required to achieve a protected database on a blockchain. It also shows the results gotten when the blockchain system was tested. The software used include the following.

### Ganache

Ganache is a private blockchain for quick creation of Corda and Ethereum distributed applications. You may create, deploy, and test your apps using it across the whole development cycle in a secure and predictable environment. There are two flavors of ganache: UI and CLI. A desktop program called Ganache UI supports both Corda and Ethereum. For Ethereum development, ganache-cli, originally known as the TestRPC, is a command-line tool.^36^^,^^37^

### MetaMask Ethereum wallet

A program or cryptocurrency wallet called MetaMask is used to communicate with the Ethereum network. Users can utilize a browser extension or mobile app to access their Ethereum wallet, which can then be used to connect with decentralized applications. Users of MetaMask can transfer and receive Ethereum-based cryptocurrencies and tokens, broadcast transactions, store and manage account keys, and securely connect to decentralized applications using a compatible web browser or the mobile app’s built-in browser. It also enables the activation of smart contracts.^38^^,^^39^

### Truffle framework

Truffle is a programming environment, testing framework, and asset pipeline. A developer can create the front ends for the apps as well as inject Smart Contracts into web apps using Truffle. With the goal of simplifying the work of developers, Truffle is a top-notch programming environment, testing framework, and asset pipeline for blockchains running on the Ethereum Virtual Machine (EVM).^40^ In this study, Truffle was used as the testing framework for the system. The implementation of the data protection system for a smart healthcare-based system using blockchain technology in this study presents several novel aspects, leveraging blockchain’s inherent properties to address critical challenges in healthcare data management, which include enhanced security and privacy, patient-centric data control, interoperability and standardization, data integrity and reliability, and efficient data management.

### Solidity

Smart contracts are written in the object-oriented, high-level programming language called Solidity. A program that regulates how Ethereum accounts behave is known as a smart contract. It is intended to target the Ethereum Virtual Machine and is influenced by C++, Python, and JavaScript (EVM). Support is provided for advanced user-defined types, libraries, and inheritance.^41^^,^^42^^,^^43^

### System requirements

System requirements are the fundamentals that a projected system demands before it can function effectively as it was designed to. The user of the system will have to meet up with the system requirement to be able to use the system successfully. These requirements include the following:

(1) Operating system: Windows 10 and above versions
(2) Processor Memory (RAM): minimum of 4 GB
(3) Processor Speed (RAM): minimum of 2.0 GHz
(4) Processor: Intel(R) Core (TM) i3-6100U CPU @ 2.30 GHz: minimum of 2.0 GHz
(5) Installed RAM: 8.00 GB (7.89 GB usable)
(6) System type: 64-bit operating system, x64-based processor
(7) Storage: Local Disk NTFS 297 GB: minimum 20 GB

### System result

**Figure 1:**
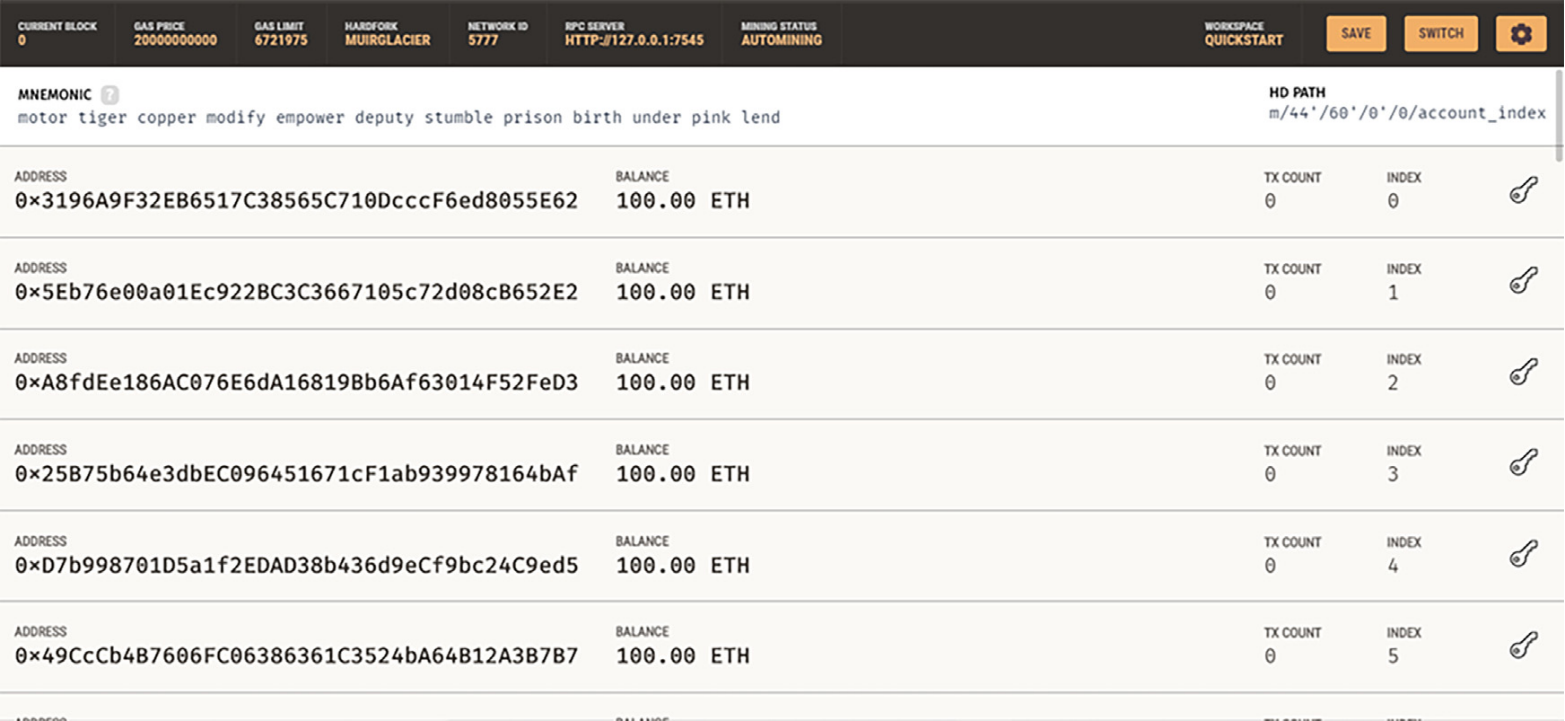
Default page of the Ganache test network

In this study, the application Ganache was firstly downloaded and installed on the computer system using the Google Chromium; it is controlled by the MetaMask Ethereum wallet, then dependencies are installed using the terminal, in this case the Microsoft terminal. Dependencies installed are Node.JS by using the command node -v. Truffle is also installed using the CMD and the command line npm install -g truffle@5.05. The Ganache platform is a test network that is hosted locally on the PC. The Ganache test network is started up with Fifty (50) active accounts with 100 ether each. “Ether” is short for Ethereum, and it is the default crypto currency on the Ganache platform. It is proposed as a medium for exchange on this system (that is when a transaction takes place or a smart contract). The Ganache is then connected with MetaMask, but first Google Chromium was downloaded to enable the MetaMask extension to run smoothly. Once the Google Chromium is downloaded and installed, the MetaMask extension is added to the chromium browser, making sure it is in the list of extensions on the browser. A fox icon can be seen in the top right-hand side of your Google Chromium when it is installed. Figure 1 shows the Ganache Default setting page.

**Figure 2:**
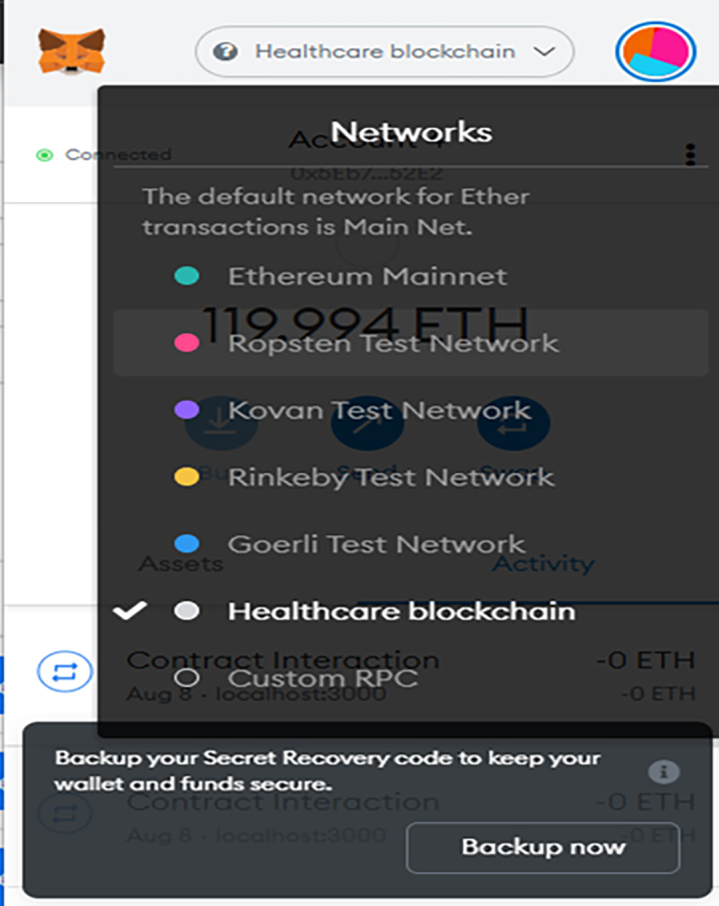
MetaMask browser extension with a new network added

Once the MetaMask is connected to the Ganache platform, a default account is auto-generated called Account 1. This is shown in Figure 2. Ganache has an IP of 127.0.0.1 and a port of 7545. In MetaMask, there is a dropdown menu above “Account 1” that currently says “main Ethereum Network,” from there select Custom RPC. Then under New RPC URL input http://127.0.0.1:7545, click save to save the state. The new network is added to the MetaMask platform and renamed to “Healthcare Blockchain.”

**Figure 3:**
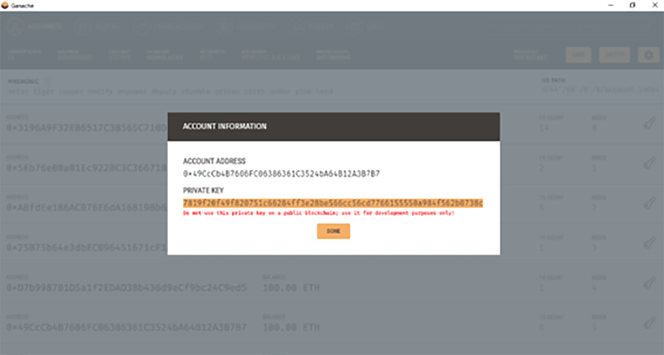
MetaMask browser extension showing a private key

Ganache wallets can now be imported to MetaMask by clicking the top-right icon and selecting import account. This option would ask for the private key to import. The private key can be gotten from Ganache by clicking on the key icon on the right-hand side of the wallet interface. It is then copied and pasted into MetaMask import, and a Hundred (100) ethers is ready to be used for transactions on the private Ganache blockchain. Multiple accounts can now be added to the Ganache blockchain. Figure 3 shows a MetaMask browser extension with a private key.

### Smart contract initiation

The Smart contract was written in JavaScript and is deployed on the blockchain using Truffle. Truffle is able to handle the written smart contract because of the previously installed dependencies. The smart contract written with JavaScript was tested to simulate client-side interaction with our smart contract. The test was written in JavaScript with the Mocha testing framework and the Chai assertion library. All these are available in the Truffle framework.

The test does two things:

(1) It checks that the name was set when it was deployed.
(2) It also checks that the smart contract has an address and was successfully deployed to the network.

Solidity allows the creation of unique data structures, with any arbitrary attributes. In this system, it was done by creating a patient struct. It stores all the attributes of a patient that would include the name, address, and diagnosis.

### The data protection front-end

**Figure 4:**
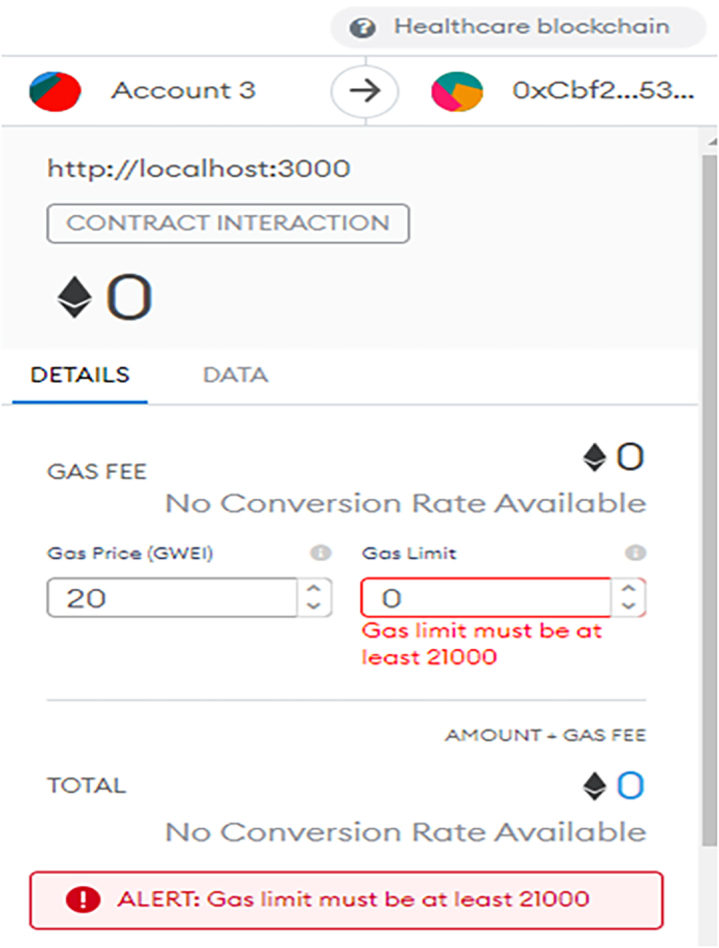
MetaMask interface showing a transaction has been requested

**Figure 5:**
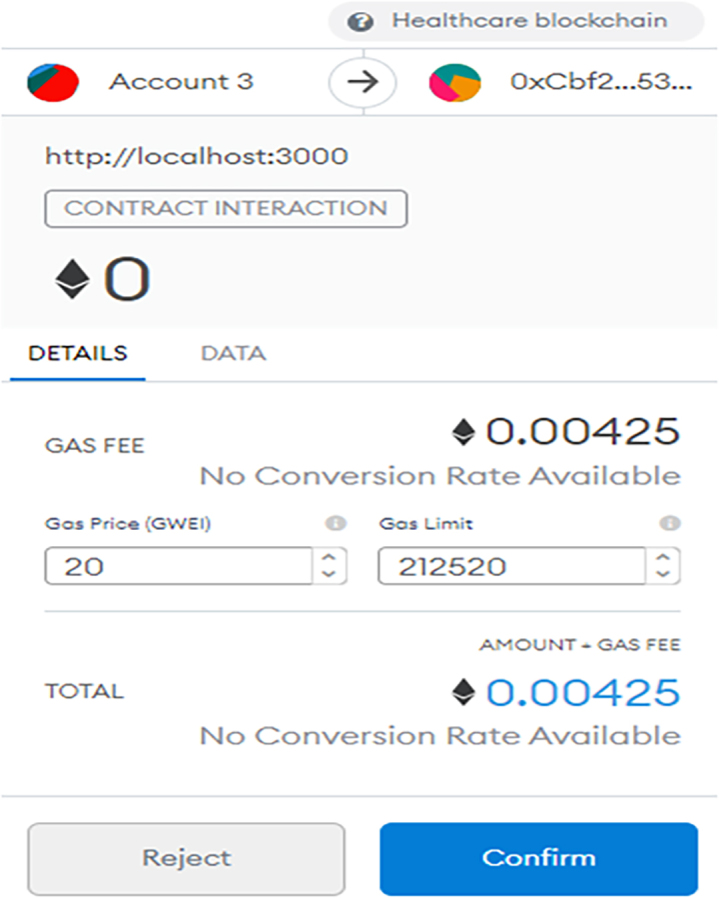
MetaMask interface showing a transaction should confirm

**Figure 6:**
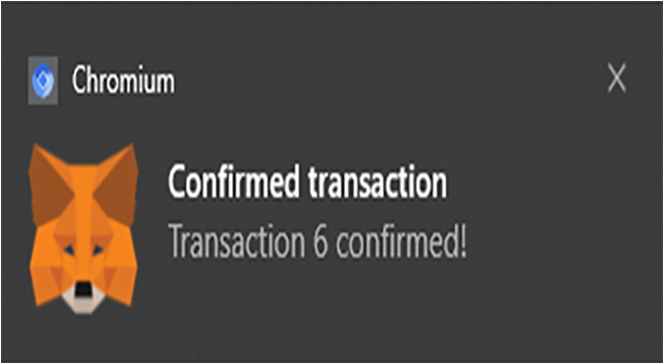
MetaMask interface showing a transaction is confirmed

The Ganache platform and the already connected MetaMask needs to be linked up with the front end, to enable user access. The development server will start running after using the command “npm run start.” React.js was used for building the interface, whereas the Bootstrap was used for creating UI elements. Once a transaction is requested (that is a smart contract is initiated), the MetaMask pops out to request that the user confirms and shows the required gas fee for the exact transaction. A typical transaction request is shown in the MetaMask interface of Figures 4, 5, and 6, which shows a confirmation interface for a transaction.

**Figure 7:**
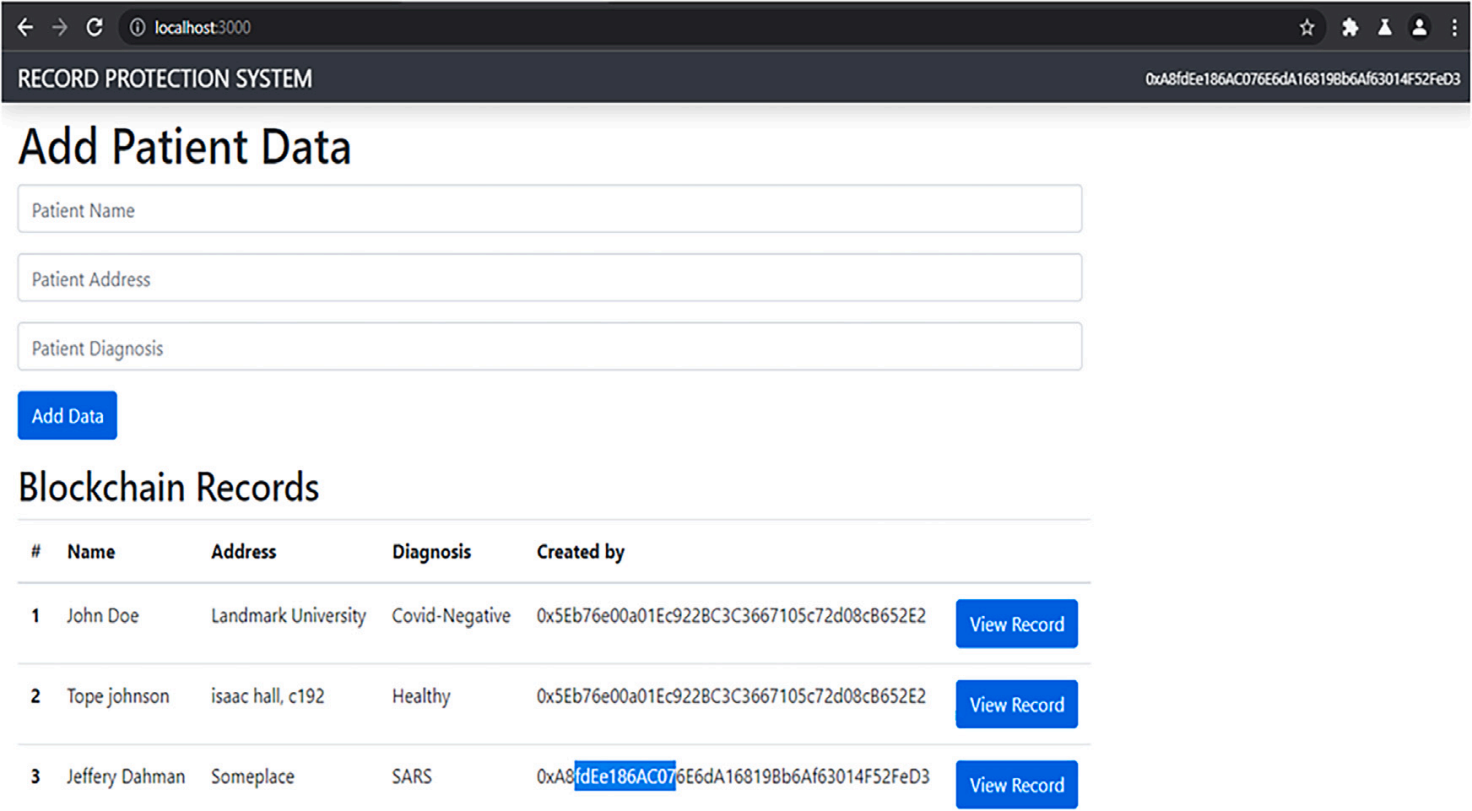
Front-end of the EHRM interface

**Figure 8:**
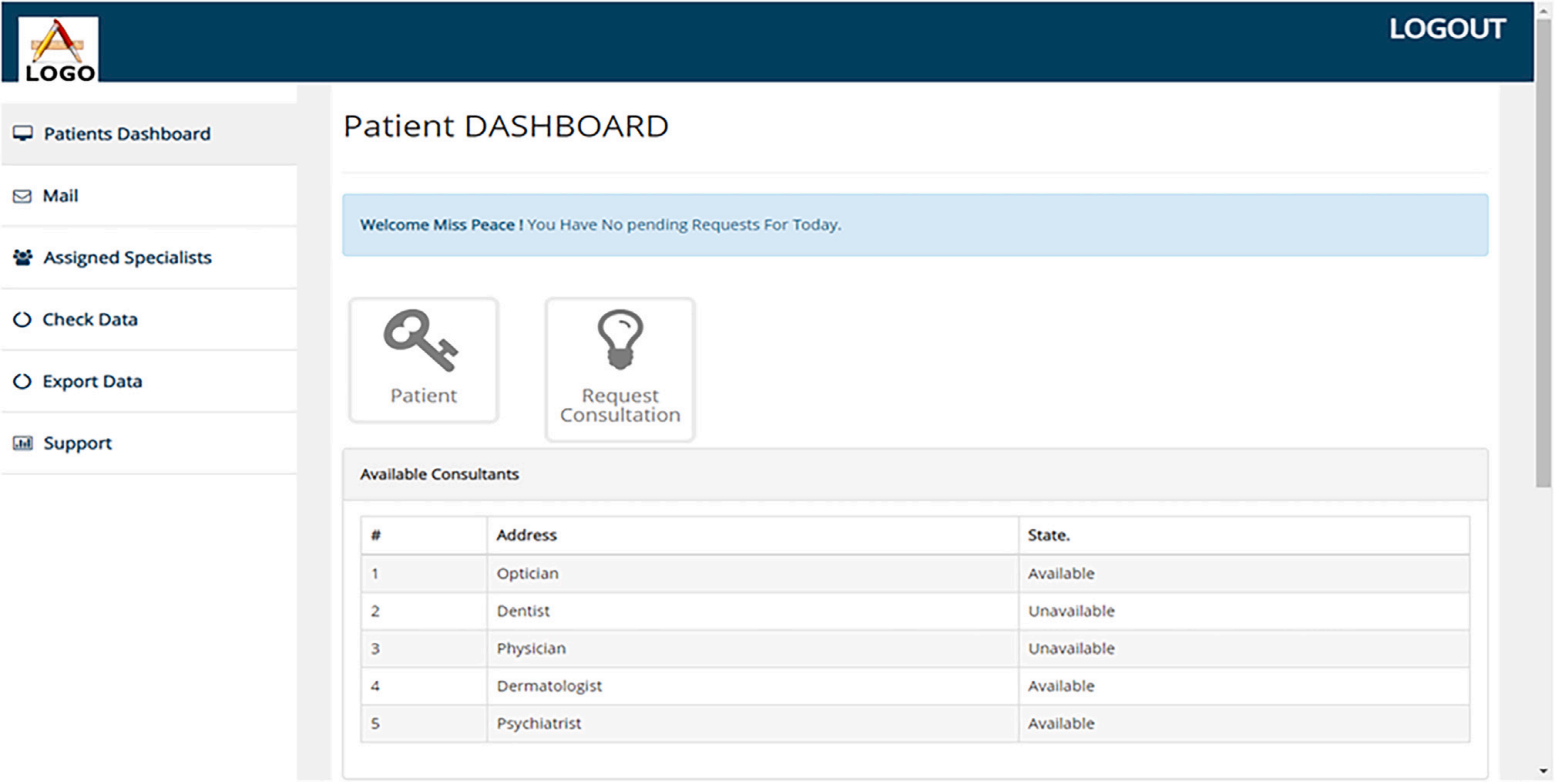
Front-end of the patient interface

**Figure 9:**
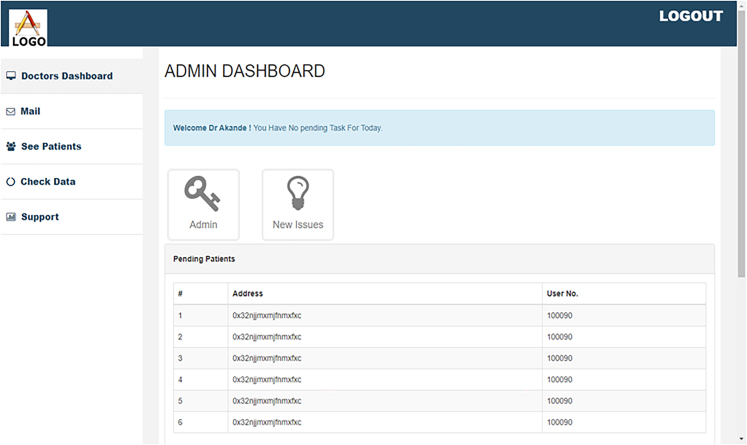
Front-end of the doctor’s interface

**Figure 10:**
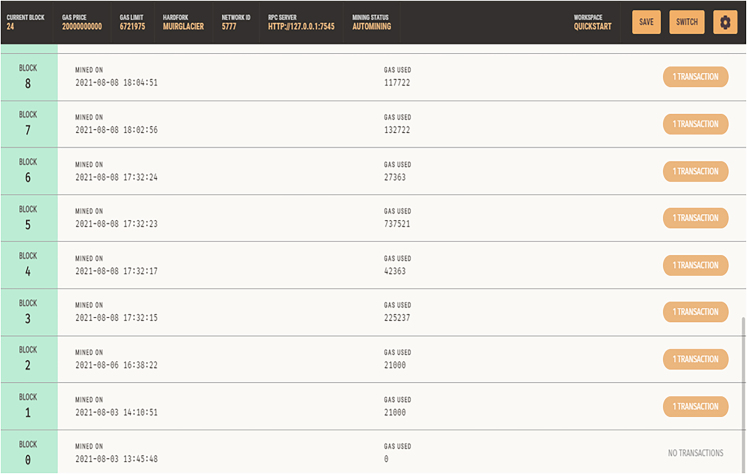
Ganache blocks: Block 0 indicates genesis block

**Figure 11:**
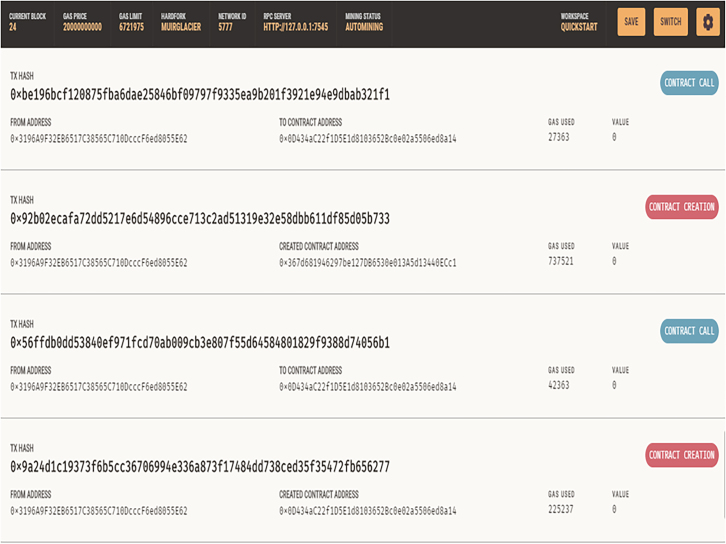
Ganache transactions: smart contract initiated and approved

The Doctors and Nurses interfaces are created for easy interaction with the blockchain. They are created and written in Bootstrap. The patient’s interface was also created along with the EHRM frontend interface as shown in Figures 7, 8, and 9. Figure 10 shows the creation of a Ganache blocks on the chain. Figure 11 shows Ganache smart contract initiation and approval.

### Latency result and comparison

The result obtained by the proposed system and a comparison with similar systems is presented in Table 1. The table shows the latency gotten for each number of nodes respectively. The execution time as the number of hospitals using the IPFS increases was also shown in Table 2. Table 1 shows that the proposed system was a bit slower for about 100 nodes. However, there was an improvement as the node increased from around 300 nodes to 500 nodes. The increase in the number of nodes would signify an increase in patients that can be traced to an increase in the number of hospitals. Low latency indicates better performance, as data can be transmitted more quickly, or in the case of blockchain, the time taken to validate and confirm a transaction on the network is quick. Hence the noted increase in the processing time as the number of hospitals increased in Table 2. Our blockchain-based data protection system for the smart-healthcare-based system improved with the network size growth from 300 to 500 nodes. More nodes strengthened and distributed the blockchain, improving data integrity and security. By adding nodes to the consensus process, the system became more resistant to 51% attacks, improving security. The larger number of nodes improved the network’s fault tolerance and capacity, making healthcare data exchanges more reliable and efficient. The extension sped up verification, improving data processing and system responsiveness this in accordance with.^44^ The smart healthcare system’s blockchain-based data protection architecture became more secure, resilient, and efficient with 500 nodes.

**Table 1:** Comparison of the proposed system with similar existing systems

Author (s)	Latency for each number of nodes		
	100 nodes	300 nodes	500 nodes
Ismail et al.^30^	2.3 s	2.7 s	2.85s
Ismail et al.^45^	4.0 s	6.0 s	–
Hang et al.^28^	–	2.71 s	2.82s
Proposed system	2.5 s	2.65 s	2.82s

**Table 2:** Execution time in relation to the number of hospitals

No of hospital	Execution time (mins)
10	1
25	1.6
30	2.2
35	3.2
40	4.0

### Limitations of the study

A blockchain-based smart healthcare system presents significant potential for data security; however, its limitations such as scalability, privacy, regulatory compliance, and cost must be resolved prior to widespread adoption in healthcare systems. Ongoing research and development, coupled with collaboration among stakeholders (healthcare providers, regulators, blockchain specialists), will be essential for addressing these difficulties.

### Conclusion

Data protection and confidentiality is very important in the healthcare system, and with the help of the implemented data protection system, this can be achieved. This study proposes patient’s data storage on the blockchain network for easy access and to increase data ownership. In the system, patient’s data are added to the blockchain by using MetaMask and stored on the blockchain test network using the Ganache platform. The system performed well on testing, and it was able to allocate a block to a node and store the patient’s data on the wallet address/block. Low latency indicates better performance, as data can be transmitted more quickly, or in the case of blockchain, the time taken to validate and confirm a transaction on the network is quick. It provided a secure way for patient’s data to be viewed by the patient or the doctor/nurse.

## Resource availability

### Lead contact

Further information and requests for resources should be directed to and will be fulfilled by the lead contact, Jide Kehinde Adeniyi or the corresponding author Sunday Adeola Ajagbe (adeniyi.jide@lmu.edu.ng or saajagbe@pgschool.lautech.edu.ng).

### Material availability

The study did not generate new materials.

### Data and code availability

• All data can be obtained from the lead contact, provided the request is reasonable. The code related to the algorithm can be accessed by reaching out to the lead contact.
• Any additional information required to reanalyze the data reported in this paper is available from the lead contact upon request.

## Acknowledgments

The author acknowledge the support received from the Computer Science Department, 10.13039/501100016201University of Zululand, Kwadlangezwa, South Africa and the Computer Science Department, Landmark University, Omu-Aran, Nigeria.

Funding statement: the author receives no fund for the project but the project is supported by 10.13039/501100016201University of Zululand, Kwadlangezwa, South Africa.

## Author contributions

Conceptualization, writing—original draft, and software, J.K.A.; original draft, project administration, resources, and methodology, S.A.; software and writing—review and editing, A.E.A.; project administration, resources, and writing—review and editing, K.I.A.; data curation and formal analysis, A.A.; acquisition APC, review and editing, and project supervision, M.O.; validation and visualization, I.O.

## Declaration of interests

The authors declare no competing interests.

## STAR★Methods

### Key resources table

**Table:** 

REAGENT or RESOURCE	SOURCE	IDENTIFIER
**Deposited data**		

Data used for experiment in this paper	This paper	**–**

**Software and algorithms**		

Genache	Truffle suite	https://Archive.trufflesuite.com/truffle
Ethereum v1.14.12	Vitalik Brutterin, Gavim Wood	https://geth.ethereum.org
Truffle framework	Npm	https://npmjs.com/package/truffle
Solidity	Solidity	https://soliditylang.org/

### Method details

This study presents a data protection system using blockchain technology. The system increases confidentiality between patient and health personnel. The blockchain validates who can view each data on the blockchain. In the proposed system, the health record manager/Admin collects the Electronic Health Records (EHR) from the hospitals database and transfers them to the blockchain. The doctors, nurses, pharmacists and patients would represent the nodes on the blockchain and they would be the ones to verify when a new block is added to the blockchain.

The system starts by capturing the information of the patients and doctors. This is done by the patient or doctor at the Registration Centre (RC). The RC assigns a private key with an ID to the user (patient or doctor) and the assigned key and ID is sent to the Administrative Unit (AU). For either the patient or the medical personnel (doctor, nurse or pharmacists) to use the EHR, the individual must be authorized using their ID and key. If the authentication is successful, the user will be able to download data (health information) from the EHRs Manager; if not, a penalty will be imposed on the particular ID. If authentication is successful, the authorized user can access the blockchain's healthcare data.

To create the blocks in the chain, Genesis is proposed. Each block also contains the name, address and health record of a patient. Blocks can only be added by the doctors. The system uses a permissioned blockchain which is created using the Ganache together with Meta-mask (to connect the ganache to front-end). A central Interplanetary File System (IPFS) is proposed to store the previously collected data on the blockchain. This technology is also used on the BitTorrent protocol and it involves breaking up files into shards and storing them in multiple instances on the computers of blockchain nodes. The IPFS is central to all medical institutions. Request to view or add blocks to the chain is made at any health institution by the appropriate individual or patient to the HRM. The HRM of the concerned institution then reaches out to the centralized IPFS for the appropriate data. The block diagram of the proposed system is shown in figure below.

**Figure:**
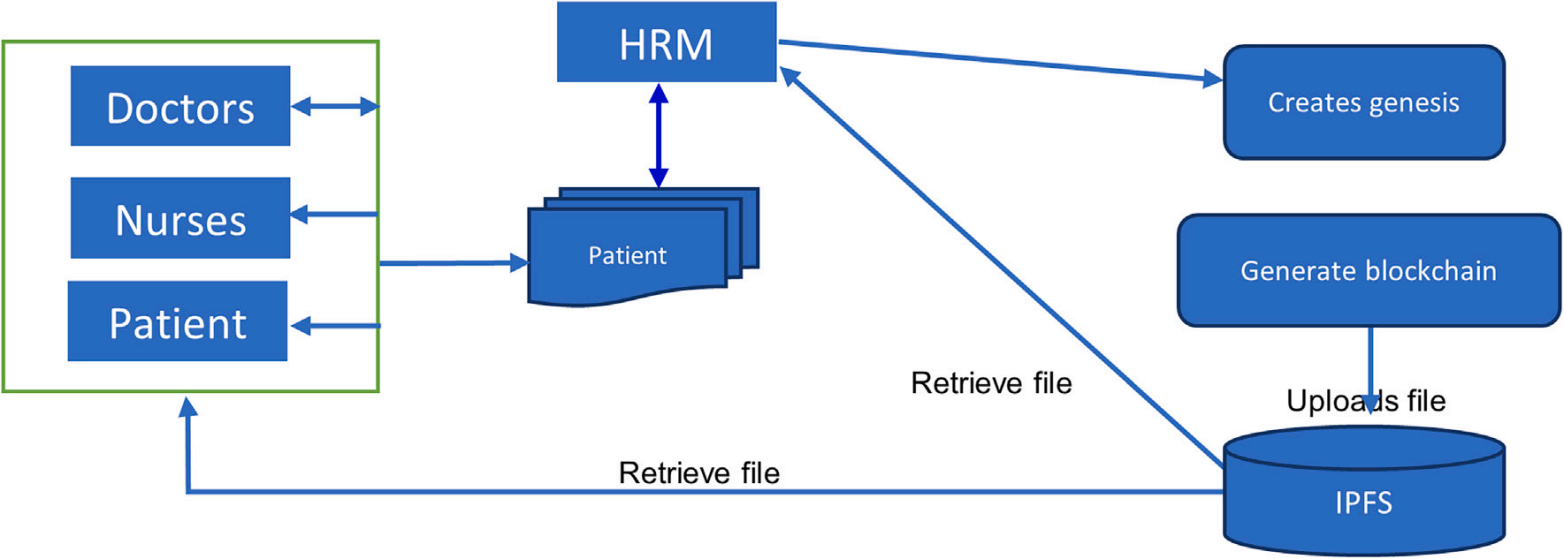
The system block diagram

#### Registration center

The registration centre is used to allocate public keys to the patient’s & medical staffs from the existing healthcare’s database. Each patient and medical staff gets a generated username and password for the them, which they can use to login into the block-chain frontend. Once the user logs-in (patient & medical staff), he/she can see the data on the blockchain based on the level of permission and request/approval from the Electronic Health Records Manager (EHRM). Using their own public keys, the registration center would also determine the identities of the doctor (DI) and patient (PI). The unique patient's or the medical staff's specific single identity and public key are then sent by the Registration Centre (RC) following a successful computation. The administrative unit receives the patient's and the medical staff's identities, which it will use to verify them when they want to get or see data from the Blockchain.

#### Electronic Health Records (EHRs) manager/ ADMINISTRATION UNIT

A patient sends a request to the EHRs Manager whenever they want to perform a transaction (patient addresses) on the blockchain or retrieve history from the blockchain. The medical staffs also go through a similar process. Every time a request is made by the patient or medical staff, the EHR manager requests the requester's public key.

The administrative unit receives the public key after it has been supplied and verifies it there. It determines whether or not the requester has permission to retrieve data from/to Blockchain using the same public key. Via a smart contract from the policy list, the administration unit verifies the requester's public key. The Interplanetary File System (IPFS), which stores data on a blockchain as key value pairs, receives an encrypted transaction from the EHRs Manager when a patient or a member of the medical staff has been successfully confirmed.

The information is distributed throughout a network of nodes or computers in 256 KB chunks. Each piece of information on IPFS has a unique hash ID. When someone requests data, they don't actually request the file itself but rather the data's hash ID.

With no need for a mediator, EHRs Manager links to Smart Contracts (SC) in a clear and conflict-free manner. While the administration unit receives the patient's and medical staff's public key and ID provided by the RC (Registration center). The Administration Unit controls all operations and transactions on the IPFS by accepting or denying.

The administration unit then confirms the requester's access privileges using the public key from the policy list after receiving a new transaction from the EHRs manager along with the user public key. When the public key is validated in the smart contract's policy list, the requester is given access to the requested data; otherwise, the request is refused and deleted from the Blockchain network.

#### Smart contract (SC)

A Smart Contract (Crypto Contract) is a computer software that legally and effectively regulates the exchange of virtual currency between a group of people under predetermined circumstances. By executing the contract, a smart contract functions similarly to a traditional contract. These are the programs that function exactly as their creators intended them to (when they were developed or changed). Similar to how traditional contracts must be enforced by law, smart contracts must be enforced by code. Manager/Administration Unit of EHRs can interact with smart contracts.^34^^,^^35^ In this system smart contract was used to initiate a new addition by the doctor to the patients record.

#### Interplanetary file system

A distributed file system can store and share data using the Interplanetary File System (IPFS), a protocol and peer-to-peer network. Each file in a global namespace connecting all computing devices is uniquely identified by IPFS via content-addressing. In order to update the hash table, it also saves the created hash. All of the storage nodes in this study are IPFS-based, and the hospital or health center's patients and medical staff maintain the IPFS system. The address is derived from the file's content using a content addressing approach. Each file is hashed into a unique hash string that serves as the file's identifier. Anyone can access the entire file saved in IPFS by using the file's blockchain hash. IPFS makes it feasible to efficiently distribute enormous amounts of data. When a new transaction occurs in the proposed system, the EHRs manager first confirms it in the administrative unit under the policy list. The transaction is divided into 256 KB chunks and sent across a network of nodes or computers since it needs to be stored after being verified. A hash is computed and stored in a table known as a hash table prior to storage. The next secure transaction is sent to a collection of transactions known as the transaction pool. There are two different types of transactions in this pool.

1) A transaction that the Blockchain would attach.
2) Transactions that mining can extract from the Blockchain.

#### Hash table

The hash table is used to store the calculated hash of all the approved transactions which would append to the Blockchain network. In our proposed system when both the patient’s and medical staff agree on the appending of the transactions to the Blockchain, they send their agreement along with the signature which proves that a particular transaction will be available for the future use when needed.

#### Transaction pool

A list of all the unconfirmed transactions may be seen in the transaction pool. The contents of the transaction pool are accessible and can be seen in real time because they are stored on a unique device. As a transaction is entered onto the blockchain, all nodes instantly record, verify, and settle the transaction's data. A confirmed change that is recorded on one ledger is also recorded simultaneously on all other copies of that ledger. The transaction pool can be split into two categories: transactions that need to be retrieved and truncations that need to be saved on the blockchain. Under my suggested method, the miners (EHRM/Admin units) are in charge of placing the transactions in a block, which is subsequently added to the Blockchain network after being verified.

### Quantification and statistical analysis

There are no quantification or statistical analysis to include in this study.
